# Polyisoprene-Silica Nanoparticles Synthesized via RAFT Emulsifier-Free Emulsion Polymerization Using Water-Soluble Initiators

**DOI:** 10.3390/polym9110637

**Published:** 2017-11-22

**Authors:** Dusadee Tumnantong, Garry L. Rempel, Pattarapan Prasassarakich

**Affiliations:** 1 Department of Chemical Technology, Faculty of Science, Chulalongkorn University, Bangkok 10330, Thailand; kid_dee_t@hotmail.com; 2 Department of Chemical Engineering, University of Waterloo, Waterloo, ON N2L 3G1, Canada; grempel@uwaterloo.ca

**Keywords:** RAFT polymerization, emulsifier-free emulsion, nanoparticles, nanocomposites, water-soluble initiator

## Abstract

Polyisoprene-silica (PIP-co-RAFT-SiO_2_) nanoparticles were prepared via reversible addition–fragmentation chain-transfer (RAFT) emulsifier-free emulsion polymerization using water-soluble initiators, 4,4′-Azobis (4-cyanopentanoic acid) (ACP) and 2,2′-Azobis (2-methylpropionamidine) dihydrochloride (V50). The particle size of emulsion prepared using ACP initiator was smaller than that using V50 initiator because the V50 initiator was more active toward decomposition than the ACP initiator. A high monomer conversion (84%), grafting efficiency (83%) and small particle size (38 nm) with narrow size distribution were achieved at optimum condition. The PIP-co-RAFT-SiO_2_ nanoparticles exhibited core–shell morphology with silica encapsulated with polyisoprene (PIP). The new PIP-SiO_2_ nanoparticles could be applied as effective filler in rubber composites that possess good mechanical and thermal properties.

## 1. Introduction

Emulsion polymerization is an effective method because it provides environmental-friendly process, the heat of reaction is easily released during the polymerization and the viscosity of medium remains close to that of water during the polymerization [1,2,3]. Nevertheless, controlled/living radical polymerization (CRP) has received attention for producing polymers with controlled molecular weight, narrow polydispersity of molecular weight and complex macromolecule architectures [4,5]. Among CRP techniques, reversible addition–fragmentation chain-transfer (RAFT) polymerization is applicable to a wide range of monomers in various conditions, and does not require a catalyst [6,7]. For RAFT polymerization process, the RAFT agent (thioester) is responsible for controlling chain growth [8,9]. The original RAFT functionality is retained at the end of chain when diblock structures are formed using RAFT controlled polymerization. Therefore, water-soluble polymers, for example, poly(acrylic acid) [10,11,12], poly(ethylene oxide) [13,14], poly(sodium acrylate) [15], and poly(styrene sulfonate) [16,17], were used for cooperating with the RAFT agent. This chemical structure was called the macro-RAFT agent, which was achieved in the emulsifier-free emulsion polymerization [13,17]. In the absence of emulsifier, the interaction between the RAFT agent and emulsifier (low colloidal stability, poor control of molecular weight, slow polymerization rate, etc.) was improved and it was also an environmentally desirable choice for preparation of polymer particles at low impurity content [18,19]. Previously, RAFT emulsifier-free emulsion polymerization of styrene was investigated in the presence of sodium acrylate (comonomer) and dibenzyltrithiocarbonate which resulted in a stable emulsion with a narrow particle size distribution [15]. The water-soluble poly(acrylamide)-based macro-RAFT agent was also used in RAFT emulsion polymerization of styrene without any surfactant, resulting in a small particle size with a narrow particle size distribution [18]. In addition, the preparation of n-butyl acrylate using poly(ethylene oxide)-based trithiocarbonate as a chain transfer agent presented the well-controlled polymer chains and a high conversion [14]. Besides, various molecular weight poly(styrene sulfonate)-based macro-RAFT agents were used in emulsifier-free emulsion polymerization of styrene and the macro-RAFT agent size affected the self-assembly, colloidal stability and particle size distribution [17].

Organic–inorganic nanocomposites consist of polymers and nanoparticles including montmorillonite clay [20], titanium dioxide [21], graphene [22,23] and silica [24,25,26]. Silica is an important inorganic nanofiller in composite preparation and widely used as effective reinforcement component in improving mechanical properties of polymers [27]. Due to particle agglomeration, the hydrophilic silica surface is usually modified with silane coupling agents to enhance adhesion between the silica filler and the polymer [28,29,30]. The silanol groups on the silica surface were attached by the coupling agent to form functionalized alkyl chain and then reacted with the polymer. It has been reported that the modified nanosilica was added in many polymer matrix such as poly(dimethylsiloxane)-silica (PDMS-SiO_2_) [31], polyisoprene-silica (PIP-SiO_2_) [32], styrene butadiene copolymer-silica (SBR-SiO_2_) [33], and polybutadiene-silica (PB-SiO_2_) [34].

RAFT emulsion polymerization has been applied to synthesize poly(methyl methacrylate) (PMMA)/silica nanoparticles with functionalized silica surface for improving the silica dispersion and the mechanical and thermal properties of nanocomposites [28]. Furthermore, the silanol group on the silica surface could be modified by the condensation reaction of 2-butyric acid dithiobenzoate (RAFT agent) and 3-glycidyloxypropyltrimethoxysilane [35]. The RAFT agent anchored on the silica surface reacted with styrene via RAFT polymerization resulting in good controlled molecular weight. Our previous work reported that the polystyrene (PS)-silica nanoparticles had been successfully prepared via RAFT emulsion polymerization using poly(styrenesulfonate-sodium)-RAFT agent (macro-RAFT agent) as emulsifier and modified silica and the small particle size (23–56 nm) with narrow particle size distribution (1.2–1.3) and high conversion (≥99.9%) were achieved [30].

In this work, PIP-co-RAFT-SiO_2_ synthesis via RAFT emulsifier-free emulsion polymerization was studied to find the appropriate initiator type and macro-RAFT agent to initiator ([R]:[I]) weight ratio. The PIP-co-RAFT-SiO_2_ nanoparticles used as novel fillers in natural rubber (NR) composite was investigated, and the physical properties of NR/PIP-R-SiO_2_ composites were reported.

## 2. Materials and Methods

### 2.1. Materials

Nano-SiO_2_ (Aerosil 200, Degussa, Piscataway, NJ, USA) with an average particle size of 12 nm, vinyl trimethoxysilane (VTS) (Sigma-Aldrich, St. Louis, MO, USA) and ammonia solution (25% NH_4_OH, Fisher Scientific, Fair Lawn, NJ, USA) were used as received for silica modification. Isoprene monomer (IP) (Sigma-Aldrich, St. Louis, MO, USA), sodium styrene sulfonate (NaSS) (Sigma-Aldrich) and 4-cyanopentanoic acid dithiobenzoate (RAFT agent, Sigma-Aldrich) were used without further purification. 4,4′-azobis (4-cyanopentanoic acid) (ACP) (Sigma-Aldrich) and 2,2′-Azobis (2-methylpropionamidine) dihydrochloride (V50, Wako Pure Chemical Industries, Ltd., Tokyo, Japan) were used as water-soluble initiators. The methyl ethyl ketone (MEK) (Fisher Scientific) was used for the rubber coagulation. The products were washed with petroleum ether (PE) (J.T.Baker, Inc., Phillipsburg, NJ, USA) to remove residual monomer and homopolymer.

For the preparation of prevulcanized natural rubber nanocomposites, natural rubber (NR) latex (total solid content of 60 wt %), sulfur as vulcanizing agent, zinc oxide (ZnO) and zincdiethyl dithiocarbamate (ZDEC) as vulcanization accelerators were obtained from the Rubber Research Institute of Thailand, Bangkok, Thailand.

### 2.2. RAFT Emulsion Polymerization of Isoprene (PIP-co-RAFT)

The macro-RAFT agent was synthesized according to the previous work [30]. Typically, NaSS (3 g, 14.5 mmol), ACP initiator (22.8 mg, 0.08 mmol) and RAFT agent (116.2 mg, 0.4 mmol) were dissolved in deionized water (18 mL). Then, the solution was stirred and deoxygenated by bubbling nitrogen through the solution for 30 min. Afterwards, it was immersed in an oil bath at 70 °C and the polymerization was proceeded for 6 h (70–80 wt % monomer conversion). Finally, the solution was precipitated with an excess of methanol and dried in a vacuum oven at 40 °C until constant weight.

The synthesis of PIP-co-RAFT nanoparticles was carried out in a 300 mL Parr stainless steel reactor. The macro-RAFT agent and initiator were dissolved in deionized water (35 mL). The initiator concentration was kept constant at 1 wt % based on isoprene monomer (10 g). The weight ratio of macro-RAFT agent to initiator ([R]:[I]) was varied at 1:1, 2:1, 3:1, 4:1 and 5:1. The solution was fed into the reactor equipped with an impeller stirrer, a thermocouple and a feeding tube. The solution was deoxygenated by a slow stream of nitrogen gas for 1 h at room temperature, while the stirring speed was maintained at 300 rpm and then the system was heated up to 75 °C. The feeding tube was filled with the isoprene and connected with the reactor. The monomer was continuously fed into the reactor at a given rate of 0.3 mL/min controlled by a needle valve. When the addition of the monomer was completed, the polymerization mixture was aged for 15 h. Then, particle size and particle size distribution (PSD) of obtained the PIP-co-RAFT emulsion were measured. The PIP-co-RAFT latex was precipitated using excess MEK to produce the coagulated rubber. The coagulated rubber was filtered and dried in a vacuum oven at 40 °C until constant weight. The monomer conversion was determined using a gravimetric method and calculated using Equation (1):

(1) $$Monomer\;conversion\left(\%\right)=(M_{2}/M_{1})\times 100$$

where *M*_1_ and *M*_2_ are the weight of monomer reacted and monomer charged, respectively. 

The emulsion was denoted as PIP-co-R1-ACP where R1 indicated the [R]:[I] ratio of 1:1 and ACP indicated the ACP initiator.

### 2.3. Preparation of Polyisoprene-Silica Nanoparticles (PIP-co-RAFT-SiO_2_)

The nano-SiO_2_ particles modified with VTS were prepared according to the literature [32]. PIP-co-RAFT-SiO_2_ nanoparticles synthesis was performed in a 300 mL Parr stainless steel reactor. Typically, 1 g of VTS-SiO_2_ was dispersed in deionized water using an ultrasonic bath for 1 h. Then, VTS-SiO_2_, macro-RAFT agent, initiator and deionized water (35 mL) were charged into the reactor. The isoprene polymerization followed the same procedure as the synthesis of PIP-co-RAFT nanoparticles. The PIP-co-RAFT-SiO_2_ emulsion was obtained, then the particle size and PSD were measured. The latex was precipitated using excess MEK to produce the coagulated rubber. The coagulated rubber was filtered and dried in a vacuum oven at 40 °C until constant weight. The monomer conversion determined using a gravimetric method was calculated using Equation (2):

(2) $$Monomer\;conversion\left(\%\right)=[(W_{0}-W_{1})/W_{2}]\times 100$$

where *W*_0_, *W*_1_ and *W*_2_ are the weight of the coagulated rubber composite, the charged silica particles and the charged monomer, respectively.

The PIP-co-RAFT-SiO_2_ nanoparticles were extracted using PE in a soxhlet apparatus to remove the free polyisoprene for 24 h and then the samples were dried to a constant weight. Grafting efficiency determined using a gravimetric method was calculated according to Equation (3):

(3) $$Grafting\;efficiency\left(\%GE\right)=\left(W_{G}/W_{R}\right)\times 100$$

where *W_G_* and *W_R_* are the weight of grafted polymer and total weight of polymer formed, respectively.

The silica encapsulation efficiency was determined by an acid etching method. The composite sample was slowly added to an excess of HF solution and dried to determine the weight percent of residue. The silica encapsulation efficiency was calculated as follows:

(4) $$Silica\;encapsulation\;efficiency\left(\%Si\;encap\;eff\right)=(W_{ES}/W_{S})\times 100$$

where *W_ES_* and *W_S_* are the weight of encapsulated silica and the total weight of SiO_2_ in the system, respectively.

The nanocomposite was denoted as PIP-co-R1-ACP-Si where R1 indicated the [R]:[I] ratio of 1:1, ACP indicated the ACP initiator and Si indicated the modified nanosilica.

### 2.4. Characterization of Nanoparticles

The particle size and particle size distribution (PSD) of the latex were measured using a dynamic light scattering technique (DLS, Nanotrac 150 particle size analyzer, Pittsburgh, PA, USA, range: 0.8–6500 nm, angle: 180 deg) and reported as the number-average diameter (D_n_).

The chemical structures of the macro-RAFT agent and PIP-co-RAFT nanoparticles were determined by ^1^H Nuclear magnetic resonance (NMR) spectroscopy (Bruker 300 MHz spectrometer, Billerica, MA, USA). The sample solution was prepared by dissolving 20 mg of the macro-RAFT agent and PIP-co-RAFT-SiO_2_ nanoparticles in 1 mL of deuterium oxide (D_2_O) and deuterated chloroform (CDCl_3_), respectively.

The VTS-SiO_2_ and PIP-co-RAFT-SiO_2_ nanoparticles were characterized by Fourier-transform infrared (FTIR) spectroscopy (Perkin Elmer Spectrum RX I spectrophotometer, Waltham, MA, USA). Infrared spectra were recorded in the region 4000–500 cm^−1^, with a resolution of 0.5 cm^−1^.

The morphology of PIP-co-RAFT and PIP-co-RAFT-SiO_2_ nanoparticles were examined using a transmission electron microscope (TEM, JEOL JEM-2100, Peabody, MA, USA) operating at an acceleration voltage of 80 kV. The latex sample diluted 20 times with deionized water was dropped on a 400-mesh copper grid at room temperature and the grid was stained with 1% OsO_4_ prior to analysis to obtain sufficient contrast.

### 2.5. Preparation and Properties of NR/PIP-R-SiO_2_ Nanocomposites

The PIP-co-R3-ACP-Si and PIP-co-R4-V50-Si latex were selected to blend with NR latex at various weight ratios (NR/PIP-R-SiO_2_ = 100/0, 90/10, 80/20, 70/30) under a stirring rate of 450 rpm for 30 min to form a homogeneous mixture. After that ZnO (2 phr), ZDEC (1 phr) and sulfur (1.5 phr) were dropped into the mixture, which was heated up to 60 °C with constant stirring at 350 rpm for 2 h. Then the nanocomposite latex was cooled to room temperature and cast on a glass plate (9 cm × 9 cm × 3 mm). The cast sheet was dried at 70 °C for 5 h. The composite was denoted as NR/PIP-R-SiO_2_ which PIP-R-SiO_2_ indicated the PIP-co-RAFT-SiO_2_.

The mechanical properties of the NR/PIP-R-SiO_2_ composites were measured using a Universal Testing Machine (INSTRON 5566, Norwood, MA, USA) at the cross-head speed of 500 mm/min according to ASTM D412. All specimens were cut into dumbbell-type shape, and the average of three measurements of the five specimens was obtained as the representative value.

Thermogravimetric analysis (TGA) was performed on Perkin-Elmer Pyris Diamond TG/DTG (PerkinElmer, Inc., Waltham, MA, USA). The sample (10 mg) placed on a platinum pan was heated from room temperature to 800 °C at a constant heating rate of 10 °C/min with a nitrogen gas flow rate of 50 mL/min.

Dynamic mechanical thermal analysis (DMA) of NR/PIP-R-SiO_2_ nanocomposites was performed using a dynamic mechanical thermal analyzer (DMA/SDTA 861e, Mettler-Toledo Inc., Columbus, OH, USA) in a shear mode. All samples were cut into strips with dimension 4 mm × 4 mm × 2 mm. The temperature was run in the range of −80 °C to 25 °C at an oscillation frequency of 10 Hz with a heating rate of 5 °C/min. The storage modulus and the loss tangent (tan δ) curve were observed.

## 3. Results and Discussion

### 3.1. Characterization of PIP-co-RAFT and PIP-co-RAFT-SiO_2_ Nanoparticles

**Figure 1:**
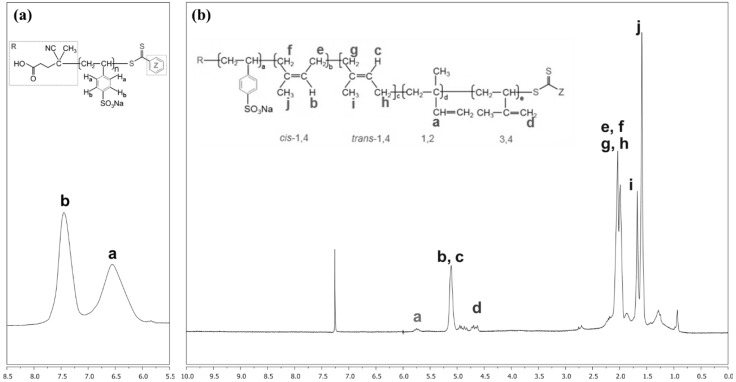
^1^H-NMR analysis of: (**a**) macro-RAFT agent; and (**b**) PIP-co-RAFT (PIP-co-R3-ACP).

Figure 1 shows NMR spectra of the macro-RAFT agent and PIP-co-RAFT nanoparticles. From Figure 1a, the macro-RAFT agent exhibits peaks a and b (6.4 and 7.4 ppm) which are assigned to aromatic protons on the phenyl rings. For PIP-co-RAFT nanoparticles (Figure 1b), the PIP has the isomeric structures with *cis*-1,4, *trans*-1,4, 3,4 and 1,2 linkages representing the diene rubber properties and the ratio of structures is estimated from the integrated peak areas of these signals. The signals in the range of 5.6–5.9, 5.0–5.5 and 4.5–5.0 ppm are attributed to 1,4-PIP, 1,2-PIP and 3,4-PIP, respectively, with the ratio of 93:3:4.

**Figure 2:**
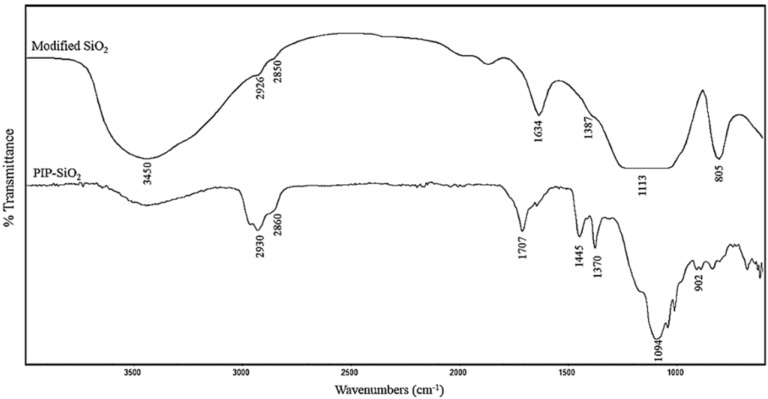
FT-IR spectra of VTS-SiO_2_ (modified nano-silica) and PIP-co-RAFT-SiO_2_ (PIP-co-R3-ACP-Si).

The FT-IR spectra of VTS-SiO_2_ nanoparticles and PIP-co-RAFT-SiO_2_ nanocomposites are shown in Figure 2. The modified nanosilica has absorption bands at 1113 and 805 cm^−1^ which are assigned to Si–O–Si groups. The peaks at 2926 and 2850 cm^−1^ correspond to CH and CH_2_ stretching of the VTS groups. The bands at 1634 cm^−1^ (C=C) and 1387 cm^−1^ (CH out of plain bending) are attributed to the double bonds of VTS. These results indicate that the coupling agent could be bonded onto silica. For PIP-co-RAFT-SiO_2_ nanocomposites, the absorption bands at 2930 and 2860 cm^−1^ are related to the methylene and methyl stretching of PIP. Furthermore, the peaks at 1707 and 902 cm^−1^ correspond to the C=C stretching and CH wag of tri-substituted olefin of PIP, respectively. The bands at 1445 cm^−1^ and 1370 cm^−1^ are attributed to the methyl deformation bands of polyisoprene. These results indicate that PIP was grafted onto the silica surface.

**Scheme 1:**
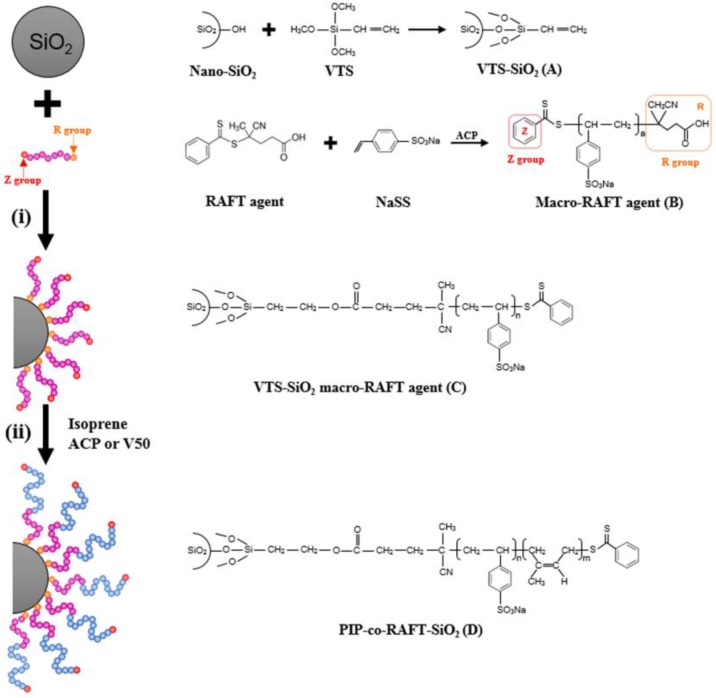
Proposed mechanism of polyisoprene-silica (PIP-co-RAFT-SiO_2_) synthesis via RAFT polymerization.

From the proposed mechanism for PIP-co-RAFT-SiO_2_ synthesis shown in Scheme 1, the nano-SiO_2_ was firstly modified by VTS coupling agent to bond onto the silica surface. The nano-SiO_2_ surface and VTS silane coupling agent generated the siloxane linkage through a polycondensation reaction to produce VTS-SiO_2_ (A). For the macro-RAFT agent synthesis, the 4-cyanopentanoic acid dithiobenzoate (RAFT agent) as the chain transfer agent reacted with the NaSS monomer to form poly(styrenesulfonate-sodium) (PSSNa)-RAFT agent (B) called macro-RAFT agent which was used as emulsifier. For the preparation of PIP-co-RAFT-SiO_2_: (i) the macro-RAFT agent (B) attached to the VTS-SiO_2_ via the addition reaction of –COOH in B to –CH_2_=CH_2_ group in A to form VTS-SiO_2_ macro-RAFT agent (C) [36,37]; and (ii) the IP monomer inserted between dithiobenzoate group and PSSNa in C to produce PIP-co-RAFT-SiO_2_ structure (D) [6,38].

### 3.2. Morphology of PIP-co-RAFT and PIP-co-RAFT-SiO_2_ Nanoparticles

**Figure 3:**
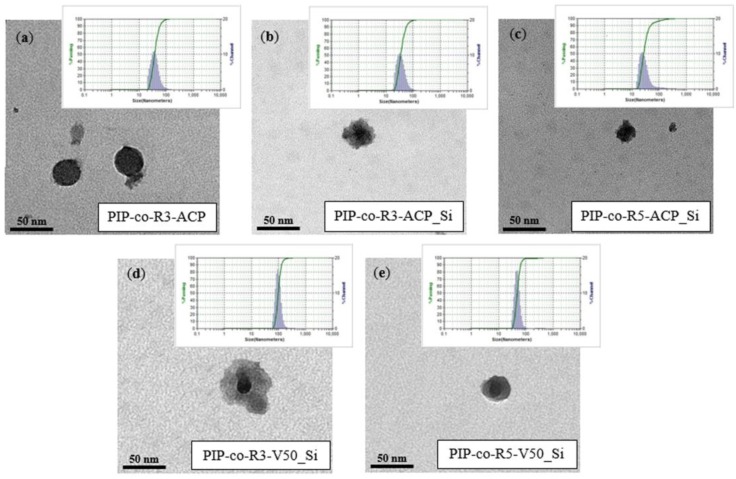
TEM micrographs of (**a**) PIP-co-R3-ACP, (**b**) PIP-co-R3-ACP_Si, (**c**) PIP-co-R5-ACP_Si, (**d**) PIP-co-R3-V50_Si and (**e**) PIP-co-R5-V50_Si.

The morphology of PIP-co-RAFT and PIP-co-RAFT-SiO_2_ nanoparticles characterized by TEM are shown in Figure 3. The TEM images of PIP-co-R3-ACP emulsion (Figure 3a) presented the spherical PIP particles (without silica) with a diameter of around 37 nm. The micrographs of PIP-co-RAFT-ACP-SiO_2_ and PIP-co-RAFT-V50-SiO_2_ nanoparticles (Figure 3b–e) exhibited a core–shell structure, in which the darker area represented the nanosilica core region and the lighter area represented the PIP grafted onto the silica surface as the shell. Moreover, Figure 3 also shows that the nanoparticles were well-dispersed in the emulsion form (particle size = 25–91 nm) and a narrow PSD (1.1–1.5) was achieved for all PIP-co-RAFT and PIP-co-RAFT-SiO_2_ nanocomposites at all [R]:[I] ratios. The PIP-co-RAFT-SiO_2_ nanoparticles with core–shell morphology were successfully produced from RAFT emulsion polymerization using ACP and V50 initiators.

### 3.3. PIP-co-RAFT Nanoparticles: Effect of Initiator

The effect of initiators (ACP and V50) on the particle size, particle size distribution (PSD) and percent total solid content of PIP-co-RAFT emulsion are presented in Table 1. For ACP initiators, the particle size of PIP-co-RAFT-ACP emulsion decreased with increasing [R]:[I] ratio. This is due to that the increasing macro-RAFT agent amount representing the increasing number of hydrophilic chains lowered the chain growth resulting the decrease in particle size. It showed similar results as that of PS nanoparticle synthesis (21–45 nm) using the ACP initiator and macro-RAFT agent [30]. For PIP-co-RAFT-ACP latex, the particle size of 28-88 nm was achieved for polymerization at all [R]:[I] ratios, whereas the particle size of PIP-co-RAFT-V50 emulsion decreased with increasing [R]:[I] ratio. The polymerization was not attained at [R]:[I] ratio = 1:1 due to the coagulation of PIP-co-RAFT-V50 particles. The particle size of PIP-co-RAFT-V50 emulsion at the other [R]:[I] ratios was 59–184 nm. However, PIP-co-RAFT-ACP and PIP-co-RAFT-V50 nanoparticles at the same [R]:[I] ratio clearly showed the different particle size. PIP-co-RAFT-ACP nanoparticles were smaller than PIP-co-RAFT-V50 nanoparticles. These could be due to the different molecular structure of ACP and V50 initiators. The dissociation properties of ACP and V50 initiators are weakly acidic (–COOH) and basic [–C(=NH)–NH_2_], respectively. It can be said that the lone pair electron in the structure of V50 initiator gave the high reactivity. Interestingly, the V50 initiator decomposed at faster decomposition rate than ACP initiator due to the lower half-life decomposition temperature of V50 initiator (56 °C in water) than ACP initiator (69 °C in water) [39]. The V50 initiator that was more active than the ACP initiator could react with monomer molecules at high reaction rate in the polymerization process; thus, there were nanoparticles with greater size in the PIP-co-RAFT-V50 emulsion.

**Figure 4:**
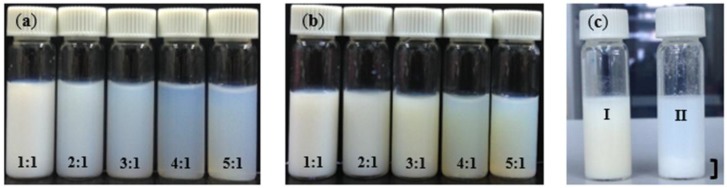
(**a**) PIP-co-RAFT-ACP emulsions; (**b**) PIP-co-RAFT-ACP-SiO_2_ emulsions prepared at various [R]:[I] ratios; and (**c**) comparison between PIP-co-R3-ACP-Si (silica encapsulated, I) and silica mixed with PIP-co-R3-ACP (physical mixing, II).

**Figure 5:**
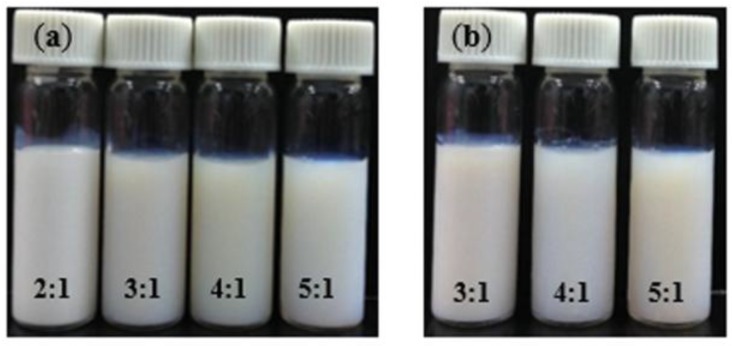
(**a**) PIP-co-RAFT-V50 emulsions; and (**b**) PIP-co-RAFT-V50-SiO_2_ emulsions prepared at various [R]:[I] ratios.

Moreover, the PSD values were in the range of 1.1–1.4, indicating that the polymerization occurred under the good growth control. For the monomer conversion, it can be seen that the monomer conversion increased with an increase in [R]:[I] ratio. Due to the fact that macro-RAFT agent played the role of a stabilizer as an emulsifier, a higher macro-RAFT agent concentration could generate more chain growth which gave more chain extension in the process resulting in a higher polymerization rate and monomer conversion. Nevertheless, PIP-co-R2-V50 and PIP-co-R3-V50 emulsion showed quite low conversion because the large particles might agglomerate and disturb the polymerization. Furthermore, the effect of [R]:[I] ratio on the characteristics of the PIP-co-RAFT-ACP and PIP-co-RAFT-V50 latex are shown in Figure 4a and Figure 5a. For PIP-co-RAFT-ACP emulsion, it can be seen that more transparent latex was produced with increasing [R]:[I] ratio. On the other hand, the appearance of PIP-co-RAFT-V50 emulsions was less transparent due to the large particle size of latex.

### 3.4. PIP-co-RAFT-SiO_2_ Nanoparticles

Effect of [R]:[I] ratio on PIP-co-RAFT-SiO_2_ synthesis using ACP and V50 initiators are presented in Table 2. The particle size of PIP-co-RAFT-SiO_2_ nanocomposites decreased with an increase in [R]:[I] ratio due to that the macro-RAFT agent amount influenced the number of hydrophilic chains. The increasing number of hydrophilic chains lowered the chain growth resulting the decrease in particle diameter. On the other hand, it can be explained that more macro-RAFT agent means better stabilization and more silica particles participating in polymerization, hence the same amount of monomer is to be distributed over a larger number of silica particles, which leads to smaller particle sizes. Similar results were observed for the synthesis of PS-SiO_2_ nanocomposites (23–56 nm) via RAFT emulsion polymerization [30]. For PIP-co-RAFT-ACP-SiO_2_ latex prepared at all [R]:[I] ratios, a particle size of 25–73 nm was achieved. However, PIP-co-RAFT-V50-SiO_2_ emulsion with particle size of 48–91 nm was synthesized for [R]:[I] ratios of 3:1, 4:1 and 5:1 except [R]:[I] ratios of 1:1 and 2:1 due to the agglomeration of particles in the polymerization system. Moreover, it was observed that PIP-co-RAFT-ACP-SiO_2_ nanoparticles exhibited the smaller particle size than PIP-co-RAFT-V50-SiO_2_ nanocomposites due to the different decomposition rate of initiators used in the RAFT polymerization. The V50 initiator had higher reactivity than the ACP initiator; therefore, the particle size of PIP-co-RAFT-V50-SiO_2_ was greater than that of PIP-co-RAFT-ACP-SiO_2_. Furthermore, the PSD was in the range of 1.2–1.5, indicating that PIP-RAFT-SiO_2_ synthesis was in the good control of emulsion polymerization.

Nevertheless, for PIP-co-RAFT-ACP-SiO_2_ synthesis, the monomer conversion increased from 64% to 91% with increasing [R]:[I] ratio and the encapsulation of SiO_2_ also increased from 18% to 74% with increasing [R]:[I] ratio except [R]:[I] ratio = 5:1. It can be explained that the macro-RAFT agent as the role of a surfactant was absorbed on the silica surface and then provided the monomers into the chain growth resulting in a higher monomer conversion and silica encapsulation efficiency. However, the high macro-RAFT agent concentration had an adverse effect on the polymerization process because the macro-RAFT agent could inhibit the growth of polymer chain resulting in lower monomer conversion and silica encapsulation efficiency. For PIP-co-RAFT-V50-SiO_2_ synthesis, the monomer conversion showed the similar result as PIP-co-RAFT-ACP-SiO_2_ synthesis but the SiO_2_ encapsulation decreased from 67% to 33% with increasing [R]:[I] ratio from 3:1 to 5:1 because of the macro-RAFT agent and the reactivity of initiator. In addition, the grafting efficiency presented the high value for both PIP-co-RAFT-ACP-SiO_2_ (86–90%) and PIP-co-RAFT-V50-SiO_2_ (82–96%).

The characteristics of the PIP-co-RAFT-SiO_2_ latex prepared using ACP and V50 at various [R]:[I] ratios are shown in Figure 4b and Figure 5b. As [R]:[I] ratio increased, PIP-co-RAFT-ACP-SiO_2_ latex became more transparent due to decreasing particle size (24.8–73.1 nm) while PIP-co-RAFT-V50-SiO_2_ emulsion did not significantly change in transparency because of their large particle sizes. Thus, PIP-co-RAFT-SiO_2_ nanocomposites with monodispersion of silica in the latex could be achieved by the RAFT emulsion polymerization. For comparison, Figure 4c clearly shows two-phase separation of modified silica and PIP-co-RAFT emulsion in the physical mixing system. The silica agglomeration could not be broken down by mechanical shearing due to the heterogeneity caused by hydrophilic surface [33]. Thus, the homogeneity of PIP-co-RAFT-SiO_2_ latex could be achieved by silica encapsulation that reduced silica–silica interaction and enhanced silica dispersion in polymer matrix.

### 3.5. Mechanical and Thermal Properties of NR/PIP-R-SiO_2_ Nanocomposites

NR/PIP-R-SiO_2_ composites were prepared from blending NR latex with PIP-co-R3-ACP-Si and PIP-co-R4-V50-Si emulsions at NR/PIP-R-SiO_2_ ratio of 100/0, 90/10, 80/20 and 70/30 (equivalent to 0%, 1%, 2% and 3% silica content, respectively) and the mechanical and thermal properties of the composites are presented in Table 3. For NR/PIP-R-ACP-SiO_2_ nanocomposites (addition of PIP-co-R3-ACP-Si), the tensile strengths of NR blends (23.5–24.2 MPa) were higher than that of unfilled NR (17.9 MPa). This indicated that PIP-co-R3-ACP-Si nanocomposites provided a reinforcing effect on the natural rubber with a homogeneous dispersion of silica. Moreover, the modulus at 300% strain of NR blends with PIP-co-R3-ACP-Si loading was slightly increased from 1.11 to 1.24 MPa with increasing NR/PIP-R-ACP-SiO_2_ ratio. This can be explained in that these nanoparticles helped to increase the external force resistance because of the high interaction between the nanofiller and the NR matrix. In addition, the elongation at break of NR blends increased from 871% to 972% with increasing PIP-co-R3-ACP-Si loading due to the flexible and elastic nature of polyisoprene.

For NR/PIP-R-V50-SiO_2_ nanocomposites (addition of PIP-co-R4-V50-Si), the tensile strength of NR blends at 1% silica loading (18.4 MPa) was slightly higher than the unfilled NR. However, the tensile strength of composites was decreased to 16.0 and 15.1 MPa with the addition of PIP-co-R4-V50-Si emulsion at 2–3% silica loading due to aggregation of PIP-R-SiO_2_ nanoparticles and a lower filler-rubber interaction. Furthermore, the modulus at 300% strain was gradually increased from 1.11 to 1.37 MPa and the elongation at break was increased from 871% to 935% with increasing silica content from 0–3 wt %. This indicated that the PIP-co-R-V50-SiO_2_ as nano-fillers increased the external force resistance with well dispersed fillers and the polyisoprene increased the flexible and elastic properties of NR composites. Besides, the addition of PIP-co-R3-ACP-Si emulsion showed the better mechanical properties of NR composites than that of PIP-co-R4-V50-Si emulsion. It can be noted that the particle size of PIP-R-SiO_2_ fillers had a pronounced effect on tensile strength, modulus at 300% strain and elongation at break indicating that PIP-co-R3-ACP-Si (smaller particle size) was well-dispersed in the NR latex. Nevertheless, PIP-R-SiO_2_ nanoparticles synthesized via RAFT polymerization could improve the mechanical properties of NR composites.

For comparison of NR/PIP-R-SiO_2_ and NR/PS-R-SiO_2_ nanocomposites (our previous work [30]), the change of mechanical properties with increasing silica content is remarkable. The modulus at 300% strain of NR/PS-R-SiO_2_ composites (1.8–3.0 MPa) was higher than that of NR/PIP-R-SiO_2_ composites (1.1–1.5 MPa) due to the rigid and stiff properties of PS in NR matrix. However, the elongation at break of NR/PIP-R-SiO_2_ composites (889–972%) exhibited the higher value than that of NR/PS-R-SiO_2_ composites (806–710%) and the unfilled NR because of the flexible properties and the good compatibility of PIP rubber in NR phase.

**Figure 6:**
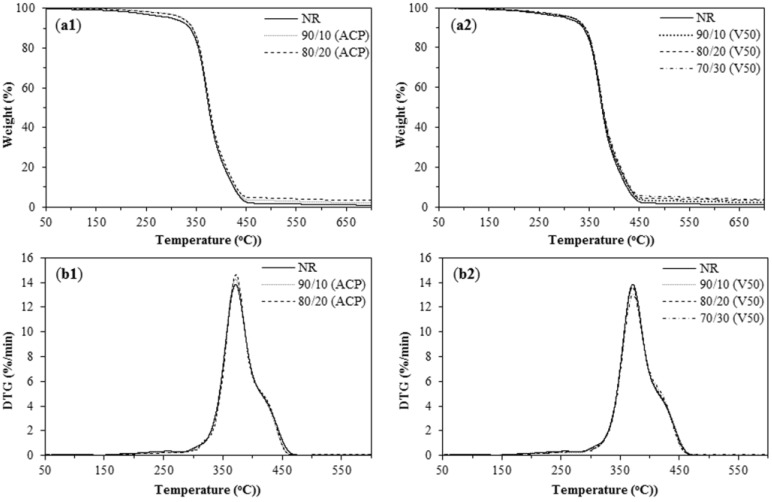
Thermal analysis of NR filled with PIP-R-SiO_2_ nanocomposites at various ratios, TGA curves: (**a1**) NR/PIP-co-R3-ACP-Si; and (**a2**) NR/PIP-co-R4-V50-Si; and DTG curves: (**b1**) NR/PIP-co-R3-ACP-Si; and (**b2**) NR/PIP-co-R4-V50-Si.

From TG/DTA analysis (Figure 6), the NR and NR/PIP-R-SiO_2_ (90/10, 80/20 and 70/30) blends show one-step polymer degradation with smooth weight loss curves. In Table 3, the initial decomposition temperature (T_id_) and maximum decomposition temperature (T_max_) of NR/PIP-R-SiO_2_ composite (addition of PIP-co-R3-ACP-Si and PIP-co-R4-V50-Si) did not significantly change because of the low silica content (1–3%).

### 3.6. Dynamic Mechanical Properties of NR/PIP-R-SiO_2_ Nanocomposites

**Figure 7:**
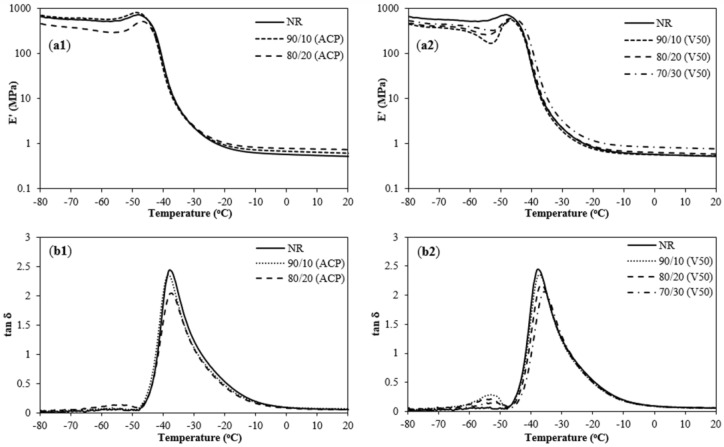
Temperature and NR/PIP-R-SiO_2_ ratio dependence of storage modulus (E′): (**a1**) NR/PIP-co-R3-ACP-Si; and (**a2**) NR/PIP-co-R4-V50-Si; and loss tangent (tan δ): (**b1**) NR/PIP-co-R3-ACP-Si; and (**b2**) NR/PIP-co-R4-V50-Si.

The elastic modulus of a material and its mechanical damping or energy dissipation characteristics as a function of frequency and temperature can be measured by DMA. The storage modulus (E′) of the unfilled NR and NR/PIP-R-SiO_2_ composites at different blend ratios are shown in Figure 7 and Table 4. At low temperature, the E′ of the NR/PIP-R-ACP-SiO_2_ composites (addition of PIP-R3-ACP-Si) at 1% silica content (814 MPa) showed the quite high values at the glassy state because of inherent semi-crystalline characteristic [40]. Nevertheless, the E′ of the NR/PIP-R-ACP-SiO_2_ composites (addition of PIP-R3-ACP-Si) at 2% silica content and NR/PIP-R-V50-SiO_2_ (addition of PIP-R4-V50-Si) at 1–3% silica content were lower than that of unfilled NR (732 MPa). The PIP-R-SiO_2_ fillers may have an unexpected influence on NR latex. Moreover, it was observed that the E′ of all samples was decreased around the transition region which was a state after the onset of a sharp reduction in storage modulus because the mobility of the polymer chains increased with increasing temperature [41]. Above the glass transition temperature, the E′_20°C_ of the NR/PIP-R-SiO_2_ composites with the addition of PIP-R3-ACP-Si and PIP-R4-V50-Si increased with increasing silica loading. It can be explained that these results were the descending parts of E′ curves and the subsequent rubbery plateau [40]. Therefore, the PIP-R-SiO_2_ nano-fillers could be used as the reinforcement in NR matrix on the descending parts. 

The tan δ or loss tangent is determined from the ratio of dynamic loss modulus (E′′) to storage modulus (E′). The tan δ of the unfilled NR and NR/PIP-R-SiO_2_ composites at different blend ratios are shown in Figure 7 and Table 4. The height of tan δ curve indicated the damping property of material. The tan δ values of NR/PIP-R-SiO_2_ composites decreased with increasing silica content (the addition of PIP-R3-ACP-Si and PIP-R4-V50-Si). Silica is a rigid material resulting in restricted mobility of the polymer chain. Therefore, the addition of PIP-R-SiO_2_ nanocomposites in the system led to reduce damping property [42]. However, the NR/PIP-R-V50-SiO_2_ blends showed two peaks of E′ and tan δ curves due to the immiscibility of the samples [43]. In addition, *T_g_* could be determined from the highest value of the tan δ curve. The *T_g_* values of the NR/PIP-R3-ACP-Si composites (at 0–2% silica content) were in the range of −39.8 to −37.6 °C while the *T_g_* values of the NR/PIP-R4-V50-Si composites (at 0–3% silica content) ranged from −39.8 to −36.0 °C. In addition, the *T_g_* of NR/PIP-R-SiO_2_ blends increased with increasing PIP-R3-ACP-Si and PIP-R4-V50-Si loading because the addition of nano-fillers gave a lower flexibility and mobility of the polymer chains.

## 4. Conclusions

Water-soluble initiators, ACP and V50, were successfully used in RAFT emulsifier-free emulsion polymerization for synthesis of polyisoprene-silica nanoparticles. For PIP-co-RAFT and PIP-co-RAFT-SiO_2_ preparation, the initiator type and the [R]:[I] ratio had a pronounced effect on particle size and monomer conversion. The particle size of PIP-co-RAFT-ACP-SiO_2_ (25–73 nm) was smaller than that of PIP-co-RAFT-V50-SiO_2_ (48–91 nm) and decreased with increasing [R]:[I] ratio. Moreover, the PIP-co-RAFT-SiO_2_ emulsion presented quite high grafting efficiency and a core–shell structure as confirmed by TEM micrographs. The efficient initiator is the key of RAFT polymerization, which the new V50 initiator is one alternative for polymer-silica synthesis compared with the common ACP initiator. 

The mechanical properties of NR/PIP-R-ACP-SiO_2_ composites were higher than that of unfilled NR indicating that the compatibility of PIP-R-SiO_2_ and NR matrix could improve the composite properties. For dynamic mechanical properties of composites above glass transition, the PIP-R-SiO_2_ fillers gave the expected reinforcement in NR. Therefore, the PIP-R-SiO_2_ nanocomposite is novel filler (at low content of 1–2 wt %) in NR latex for the film, sheet and membrane applications.

## Figures and Tables

**Table 1:** PIP-co-RAFT nanoparticles prepared at various [R]:[I] ratios.

Name	[R]:[I] ^a^	Particle size (nm)	PSD	% Total solid content	Monomer conversion (%)
**ACP initiator**					
PIP-co-R1-ACP	1:1	87.5	1.2	16.1	72.9
PIP-co-R2-ACP	2:1	54.0	1.3	17.0	77.3
PIP-co-R3-ACP	3:1	37.4	1.3	16.3	74.5
PIP-co-R4-ACP	4:1	36.3	1.4	17.8	81.0
PIP-co-R5-ACP	5:1	28.0	1.4	18.8	86.2
**V50 initiator**					
PIP-co-R1-V50	1:1	- ^b^	- ^b^	- ^b^	- ^b^
PIP-co-R2-V50	2:1	184.3	1.1	7.4	31.8
PIP-co-R3-V50	3:1	165.9	1.1	10.0	44.4
PIP-co-R4-V50	4:1	61.5	1.2	19.8	85.5
PIP-co-R5-V50	5:1	58.6	1.1	18.6	81.8

^a^ [R]:[I] = weight ratio of macro-RAFT agent to initiator. Condition: Initiator = 0.1 g, H_2_O = 35 g, Isoprene = 10 g, Temp = 75 °C, Time = 15 h. ^b^ Coagulation of PIP-co-RAFT nanoparticles.

**Table 2:** PIP-co-RAFT-SiO_2_ nanoparticles prepared at various [R]:[I] ratios.

Name	[R]:[I] ^a^	Particle size (nm)	PSD	% Total solid content	Monomer conversion (%)	% GE	% Si Encap eff
**ACP initiator**							
PIP-co-R1-ACP-Si	1:1	73.1	1.5	14.8	64.0	86.4	18.3
PIP-co-R2-ACP-Si	2:1	44.1	1.5	17.1	77.1	90.4	50.4
PIP-co-R3-ACP-Si	3:1	37.5	1.2	19.2	84.2	82.5	55.0
PIP-co-R4-ACP-Si	4:1	27.2	1.5	21.0	91.0	89.1	73.5
PIP-co-R5-ACP-Si	5:1	24.8	1.5	19.0	86.8	89.2	61.8
**V50 initiator**							
PIP-co-R1-V50-Si	1:1	- ^b^	- ^b^	- ^b^	- ^b^	- ^b^	- ^b^
PIP-co-R2-V50-Si	2:1	- ^b^	- ^b^	- ^b^	- ^b^	- ^b^	- ^b^
PIP-co-R3-V50-Si	3:1	90.9	1.5	19.5	80.4	82.2	66.5
PIP-co-R4-V50-Si	4:1	66.1	1.4	22.1	96.0	91.4	56.6
PIP-co-R5-V50-Si	5:1	48.2	1.2	16.8	48.2	96.2	33.1

^a^ [R]:[I] = weight ratio of macro-RAFT agent to initiator. Condition: Initiator = 0.1 g, H_2_O = 35 g, Isoprene = 10 g, Silica = 1 g, Temp = 75 °C, Time = 15 h. ^b^ Coagulation of PIP-co-RAFT-SiO_2_ nanoparticles.

**Table 3:** Mechanical and thermal properties of NR/PIP-R-SiO_2_ nanocomposites.

Properties	NR	NR/PIP-R-ACP-SiO_2_ (ACP initiator) ^a^		NR/PIP-R-V50-SiO_2_ (V50 initiator) ^b^		
NR/PIP-R-SiO_2_	100	90/10	80/20	90/10	80/20	70/30
SiO_2_ content ^c^ (wt %)	-	1.0	2.0	1.0	2.0	3.0
Mechanical properties						
Tensile strength (MPa)	17.9 ± 0.7	24.2 ± 2.1	23.5 ± 1.0	18.4 ± 1.6	16.0 ± 1.2	15.1 ± 2.8
300% Modulus (MPa)	1.11 ± 0.02	1.19 ± 0.02	1.24 ± 0.01	1.09 ± 0.01	1.19 ± 0.01	1.37 ± 0.08
Elongation at break (%)	871 ± 32	947 ± 08	972 ± 15	889 ± 39	891 ± 34	935 ± 77
Thermal properties						
T_id_ (°C)	349.4	348.7	348.8	348.9	348.6	348.8
T_max_ (°C)	376.7	375.6	376.7	377.0	376.6	377.3

^a^ PIP-co-R3-ACP-Si; ^b^ PIP-co-R4-V50-Si; ^c^ Silica content based on total rubber.

**Table 4:** Dynamic mechanical properties of NR/PIP-co-R-SiO_2_ nanocomposites.

NR/PIP-co-R-SiO_2_ (wt/wt)	SiO_2_ Content ^c^ (wt %)	E′_max_ (MPa)	E′_20°C_ (MPa)	tan δ	*T_g_* (°C)
100/0	-	732	0.53	2.44	−39.8
**NR/PIP-R-ACP-SiO_2_ (ACP Initiator) ^a^**					
90/10	1.0	814	0.62	2.36	−38.8
80/20	2.0	517	0.74	2.04	−37.6
**NR/PIP-R-V50-SiO_2_ (V50 Initiator) ^b^**					
90/10	1.0	584	0.55	2.36	−37.5
80/20	2.0	608	0.60	2.17	−36.9
70/30	3.0	591	0.79	2.06	−36.0

^a^ PIP-co-R3-ACP-Si; ^b^ PIP-co-R4-V50-Si; ^c^ Silica content based on total rubber.
